# MixChIP: a probabilistic method for cell type specific protein-DNA binding analysis

**DOI:** 10.1186/s12859-015-0834-3

**Published:** 2015-12-24

**Authors:** Sini Rautio, Harri Lähdesmäki

**Affiliations:** Department of Computer Science, Aalto University, Aalto, FI-00076 Finland

**Keywords:** Tumor heterogeneity, Deconvolution, ChIP-seq, Transcription factor binding sites

## Abstract

**Background:**

Transcription factors (TFs) are proteins that bind to DNA and regulate gene expression. To understand details of gene regulation, characterizing TF binding sites in different cell types, diseases and among individuals is essential. However, sometimes TF binding can only be measured from biological samples that contain multiple cell or tissue types. Sample heterogeneity can have a considerable effect on TF binding site detection. While manual separation techniques can be used to isolate a cell type of interest from heterogeneous samples, such techniques are challenging and can change intra-cellular interactions, including protein-DNA binding. Computational deconvolution methods have emerged as an alternative strategy to study heterogeneous samples and numerous methods have been proposed to analyze gene expression. However, no computational method exists to deconvolve cell type specific TF binding from heterogeneous samples.

**Results:**

We present a probabilistic method, MixChIP, to identify cell type specific TF binding sites from heterogeneous chromatin immunoprecipitation sequencing (ChIP-seq) data. Our method simultaneously estimates the binding strength in different cell types as well as the proportions of different cell types in each sample when only partial prior information about cell type composition is available. We demonstrate the utility of MixChIP by analyzing ChIP-seq data from two cell lines which we artificially mix to generate (simulated) heterogeneous samples and by analyzing ChIP-seq data from breast cancer patients measuring oestrogen receptor (ER) binding in primary breast cancer tissues. We show that MixChIP is more accurate in detecting TF binding sites from multiple heterogeneous ChIP-seq samples than the standard methods which do not account for sample heterogeneity.

**Conclusions:**

Our results show that MixChIP can estimate cell-type proportions and identify cell type specific TF binding sites from heterogeneous ChIP-seq samples. Thus, MixChIP can be an invaluable tool in analyzing heterogeneous ChIP-seq samples, such as those originating from cancer studies. R implementation is available at http://research.ics.aalto.fi/csb/software/mixchip/.

**Electronic supplementary material:**

The online version of this article (doi:10.1186/s12859-015-0834-3) contains supplementary material, which is available to authorized users.

## Background

Transcription factors are DNA-binding proteins that regulate expression of neighboring or distal genes. Most of the TFs bind only a small proportion of potential genomic sites as defined by their DNA binding domains [1]. Detailed mapping of TF binding in different cell types, conditions, diseases and among individuals is central for understanding transcriptional regulation. Three factors contribute to TF binding: sequence preference of a TF, local chromatin context, and TF coactivators and repressors [2]. Furthermore, TFs often bind to distinct subsets of potential binding sites in different cell types which results in variation in gene regulation. As an example, it is shown that on average one third of measured TF binding sites overlap between the cell lines K562 and HelaS3 [2].

In some applications, DNA-protein interactions are measured from biological samples that contain multiple cell or tissue types. Sample heterogeneity is a major confounding factor e.g. in clinical studies [3] and it can have a significant effect in TF binding profiling and it limits the conclusions that can be made about binding specificity [4]. For instance, tumor biopsy sample taken from a patient often contains unknown proportions of normal or other infiltrating cells [5]. This is problematic especially if the heterogeneous tumor sample is compared to a healthy sample or to another heterogeneous tumor sample with different proportions of contaminating cell types. As an example, different subtypes of breast cancer can be defined based on their gene expression characteristics. One of the identified subtypes is a normal-like subtype which has similar expression pattern to normal breast tissue. Nevertheless, it is argued that a normal-like subtype is only an artifact resulting from contamination of samples with normal breast tissue [6].

Manual cell separation techniques, such as cell sorting, enrichment and laser-capture microdissection can be used to isolate cell types of interest from complex tissue samples, but are expensive, time-consuming and may affect cell physiology and important interactions between different cell types [7, 8]. Computational deconvolution methods have emerged as an alternative to solve these problems. *In silico* purification allows us to process data that are measured from a mixture of several cell types by performing computational deconvolution after measuring the samples. Previously, many *in silico* purification methods have been published for gene expression [3, 4, 7–15] and DNA methylation data [5]. To our knowledge, however, there is no method to estimate cell type specific TF binding sites and cell type proportions using ChIP-seq data from heterogeneous samples.

The ChIP-seq protocol produces short sequence reads of genomic DNA that are enriched for a target of interest (here binding sites of a TF). After mapping the sequence reads to a reference genome the main analysis task is to identify TF binding sites by selecting regions with significantly large numbers of mapped reads [16]. However, it is now known that regions with high read counts do not necessarily correspond to real binding sites as read counts are affected by many biases, such as local GC content, mappability, chromatin structure and copy number variation [17]. If matching input control samples exist, such as samples generated from genomic DNA (without immunoprecipitation) or by using non-specific antibody, they can be used to estimate local background biases [18].

Given that it is important to study heterogeneous samples and knowing the aforementioned challenges and limitations, we introduce a probabilistic method for identifying cell type specific binding sites from heterogeneous ChIP-seq samples.

## Methods

### Data

#### In silico simulated data

We used publicly available ChIP-seq data from The Encyclopedia of DNA Elements (ENCODE) project [19] to demonstrate the proof-of-principle of our method. We simulated *in silico* a mixture of ChIP-seq data measuring JUND binding in two different cell lines, HepG2 and K562. Each heterogeneous sample was generated by taking, with a fixed ratio, randomly subsampled sequence reads from HepG2 and K562 samples. The selected mixture ratios of the two cell lines were 10:90, 20:80, 50:50, 80:20 and 90:10 *%*. For instance, a sample with ratio 20:80 *%* has 20 *%* of the total reads from HepG2 sample and 80 *%* of the total reads from K562 sample. All in all, we simulated three samples with each mixture proportion totaling 15 samples altogether. For each simulated mixture sample, the corresponding input control with the same mixture ratio was simulated using rabbit IgG control from the same HepG2 and K562 cell lines. All the generated ChIP samples had around 21 million aligned reads whereas input control samples had approximately 48 million aligned reads. Samples and their matching input controls are listed in Additional file 1: Table S1.

**Fig. 1:**
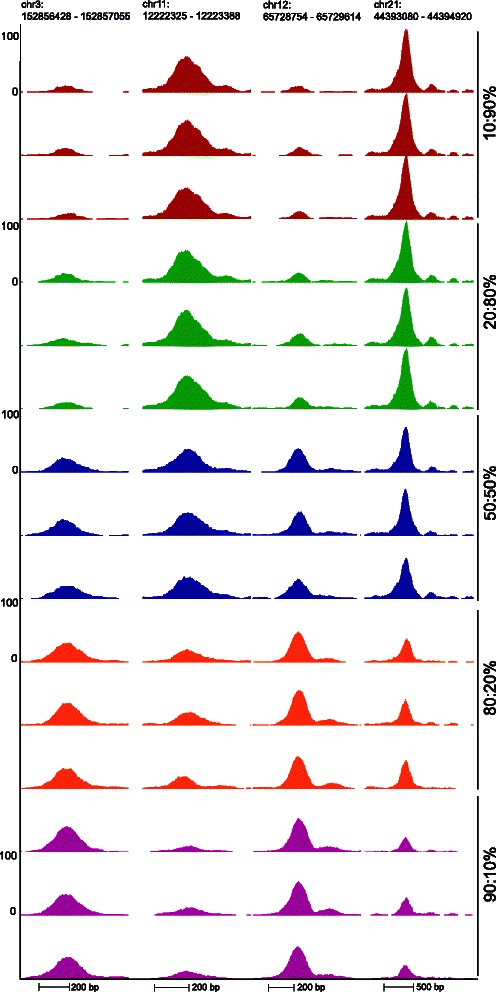
Read coverage at four representative JUND binding sites in the simulated heterogeneous samples. Signals are shown as counts per 10 million reads. The first and third columns show two binding sites where JUND is bound in HepG2 cell line but not in K562. The binding sites in the second and fourth column are JUND binding site in K562 cell line but not in HepG2. The proportions of the two cell lines in each sample are depicted on right

All simulated mixture samples were mapped to hg19 genome using Bowtie [20]. Fragment sizes were estimated with spp software using cross-correlation of positive and negative strand tag densities [21]. Next, reads were shifted according the fragment size estimates, clonal reads were removed and reads mapping to each candidate binding sites were calculated using a custom python script with HTSeq [22]. For validation purposes, true cell type specific JUND binding sites in HepG2 and K562 cell lines were detected from the original pure samples using MACS [18] with a stringent *p*-value <10^−7^. To validate the usefulness of the model 10, 000 binding sites that were found in HepG2 cell line but not in K562 were selected to represent a set of true JUND binding sites in HepG2. Similarly, the same amount of binding sites detected only in K562 cell line were selected as true JUND binding sites in K562. In addition, we selected 3160 random genomic loci which did not overlap with any of the detected JUND binding sites in the two cell lines and had on average at least three aligned reads. Thus, altogether 23, 160 candidate binding sites were used in our analysis. Figure 1 shows the simulated heterogeneous ChIP-seq signal at selected candidate binding sites.

#### Oestrogen binding data

We also applied our method to the breast cancer data from Ross-Innes *et al.* [23]. The data set consists of measurements of oestrogen receptor- *α* (ER) binding in primary breast cancer tissues and metastases. Patients were further classified in good outcome and poor outcome groups based on the tumour type. We decided to select the samples from the good outcome group patients because data from these patients correlate better with each other compared to the data from poor outcome group patients. Each patient sample has also a corresponding genomic DNA sample, which we used in the model as input controls. Samples together with their corresponding input controls and GEO accession names are shown in Additional file 1: Table S2.

**Fig. 2:**
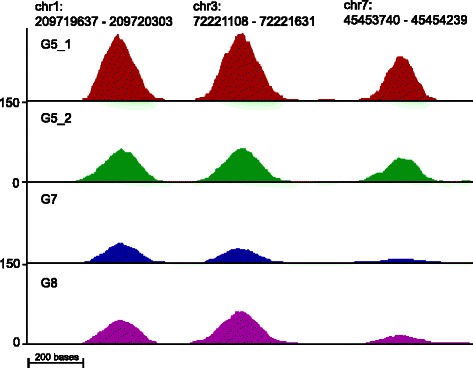
Illustrative ER binding sites in four samples used for the modeling: G5.1, G5.2, G7, and G8. Signals are shown as counts per 10 million reads

We used altogether four samples (G5.1, G5.2, G7, G8) in our model to estimate breast cancer tissue specific ER binding sites. These samples had estimated breast cancer cell percentages of 90, 90, 70 and 70 *%*. Since the ground truth of ER binding in primary breast cancer tissue is not known we used the remaining four samples to define a set of true (or most likely) binding sites. A genomic locus was considered as a true binding site if it was detected as a peak in all of the four samples G1, G2, G4 and G6. The peak calling was performed using MACS [18] with the default *p*-value threshold of 10^−5^. As a result, altogether 678 regions were selected as true binding sites. The negative set was constructed by taking random regions in the genome and discarding those which overlapped with the detected peaks from the four individual samples (G1, G2, G4 and G6) and those which had on average less than 15 reads mapped to that region, resulting in 293 loci in the negative set. Three illustrative examples of strong binding sites in the four samples used for the modeling are shown in Fig 2. Data were preprocessed in the same way as the ENCODE data set. The samples used for modeling and the *a priori* assumed proportions of breast cancer tissue in each sample are shown in Table 1.

**Table 1:** Sample list in the ER data set shown together with their *a priori* assumed tumor proportions and the number of aligned reads. Four of the samples were used in the heterogeneity modeling and rest of the samples were used to determine a set of high quality binding sites for validation purposes

Sample	Tumor percentage	Aligned Reads	Used in model
G1	> 70 %	12311074	–
G2	> 70 %	17696624	–
G4	N/A	14568154	–
G5.1	90 %	7887177	X
G5.2	90 %	16055168	X
G6	100 %	13429725	–
G7	70 %	22747279	X
G8	70 %	20227148	X

### Model

ChIP-seq data is commonly assumed to follow Poisson distribution [18]. An advantage of the Poisson distribution is that it has a single parameter *λ* which is equal to the mean and the variance of the distribution. To capture local biases in data along the genome, one of the most popular ChIP-seq peak finding algorithms, MACS [18], uses a dynamic Poisson distribution to model a local background. First, it uses a sliding window to find candidate binding regions that show enrichment of mapped sequencing reads relative to a local background model, and if enriched regions are overlapping, they are merged. The candidate sites are tested against a local background using a Poisson test, where the mean and the variance of the Poisson distribution are estimated from the global average of read counts or average read count in 1, 5 and 10 kb windows in the input control samples centred at the peak locations. We will build our cell type specific binding analysis tool on the aforementioned assumptions.

We denote the binding affinity measurement, i.e. read count, of a protein in heterogeneous sample *j*=1,…,*J* and in genomic location *i*=1,…,*I* as *y*_*ij*_. Candidate binding sites *i* which are used in our model are pre-selected; they can be for example any sites that show weak enrichment at least in one of the heterogeneous samples. The read count data across all sites and samples is denoted collectively as $\mathcal {D}$. Cell type specific binding affinities in locus *i* for cell type *t*=1,…,*T* are denoted as **x**_*i*_=(*x*_*i*1_,*x*_*i*2_,…,*x*_*iT*_) and cell type proportions in a sample *j* as **p**_*j*_=(*p*_*j*1_,*p*_*j*2_,…,*p*_*jT*_). Binding affinity measurement from a heterogeneous sample, *y*_*ij*_, is assumed to be Poisson distributed with a local parameter *λ*_*ij*_. The mean parameter *λ*_*ij*_ is a weighted average of the cell type specific binding affinities, where the weights are the cell type proportions **p**_*j*_ and *s*_*j*_, the scaling parameter for different sequencing depths of each sample

(1) $$ f(y_{ij}| \mathbf{p}_{j},\mathbf{x}_{i}, s_{j}) = \text{Poisson}\left(y_{ij}| \lambda_{ij}=s_{j} \sum\limits_{t} p_{jt} x_{it}\right).  $$

We assume the read counts in each candidate binding site *i* and sample *j* to be conditionally independent given the parameters; thus, the likelihood of the data can be written as

(2) $$ f(\mathcal{D}| \mathbf{p}, \mathbf{x})=\prod\limits_{i} \prod\limits_{j} f\left(y_{ij}| \mathbf{p}_{j},\mathbf{x}_{i}, s_{j}\right),  $$

where **p** and **x** denote all the unknowns

(3) $$ {}\mathbf{p}= \left(\begin{array}{cccc} p_{11} & p_{12} & \cdots & p_{1T} \\ p_{21} & p_{22} & \cdots & p_{2T} \\ \vdots & \vdots & \ddots & \vdots \\ p_{J1} & p_{J2} & \cdots & p_{JT} \end{array} \right), \mathbf{x}= \left(\begin{array}{cccc} x_{11} & x_{12} & \cdots & x_{1T} \\ x_{21} & x_{22} & \cdots & x_{2T} \\ \vdots & \vdots & \ddots & \vdots \\ x_{I1} & x_{I2} & \cdots & x_{IT} \end{array} \right).  $$

We place a Dirichlet distribution as the prior for cell type proportions in sample *j*, **p**_*j*_∼Dirichlet(*α*_*j*_=*w*_0_ **p**_0*j*_). Vector **p**_0*j*_ denotes the location of the distribution which can be thought of as the user’s prior information of the true cell type proportions in sample *j*, and *w*_0_ quantifies the variance of the prior which can be set based on how much the prior information is trusted. Naturally, cell type proportions in sample *j*, **p**_*j*_, as well as prior parameter **p**_0*j*_ sum up to one, i.e. $\sum _{t} p_{\textit {jt}}=1$ and $\sum _{t} p_{0jt}=1$. This makes the Dirichlet distribution a natural choice but other priors can be used as well. We set uninformative uniform priors for the unknown cell type specific binding affinities, i.e., *x*_*it*_∼Uniform(*a*,*b*), where *a* defines the lower and *b* the upper bound.

Given the read count data $\mathcal {D}$ and hyperparameters *ϕ*, our model defines a posterior distribution for the unknown parameters

(4) $$\begin{array}{@{}rcl@{}} f(\mathbf{x}, \mathbf{p} | \mathcal{D}, \phi) & \propto & \left(\prod\limits_{j} f(\mathbf{p}_{j} | \alpha_{j}) \right) \left(\prod\limits_{i} \prod\limits_{t} f\left(x_{it} | a,b\right) \right) \times  \\ & & \left(\prod\limits_{i} \prod\limits_{j} f\left(y_{ij}|s_{j} \sum\limits_{t} p_{jt} x_{it}\right) \right). \end{array} $$

We use maximum a posteriori (MAP) estimation to find the cell type specific binding affinities $\tilde {\mathbf {x}}$ and cell type proportions $\tilde {\mathbf {p}}$. Optimization of the posterior function is performed using the limited-memory modification of the Broyden-Fletcher-Goldfarb-Shanno quasi-Newton method with box constrains (L-BFGS-B) [24] (see Additional file 1 for partial derivatives of the log posterior). The optimization is performed 10 times with different initial points to avoid local optima.

Local biases can vary between cell types, samples and even replicates. Consequently, it is important to have a matching input control sample for each ChIP sample, such as genomic DNA sample or sample with non-specific antibody [17]. Input controls can contain binding site signals because TF binding sites are usually located in regions of open chromatin where fragmentation is more efficient [25]. However, our analysis suggests that input control samples contain only little or no information about the cell type proportions. Usually, the matching input control comes from the same biological source as the ChIP sample. Therefore, we assume that the matching input control sample has the same cell type proportions as the ChIP sample and decided to estimate cell type proportions, $\tilde {\mathbf {p}}$, using only the ChIP samples. In other words, the cell type proportions $\tilde {\mathbf {p}}$, estimated from the ChIP samples, are used for each of the loci in the matching input control samples. The input control signal is modeled using three different windows, similar to MACS peak finding algorithm; 1000, 5000 and 10000 base pairs around the candidate binding site. Thus, given data from the input control samples, $\mathcal {D}^{c}$, the posterior of cell type specific reads counts, **x** ^*c*^, becomes

(5) $$\begin{array}{@{}rcl@{}}  f(\mathbf{x^{c}} | \mathcal{D}^{c}, \tilde{\mathbf{p}}, \phi) & \propto &\left(\prod\limits_{i} \prod\limits_{t} \prod\limits_{r} f\left(x_{itr}^{c} | a, b\right) \right)  \\ &&\times\!\left(\!\prod\limits_{i} \!\prod\limits_{j}\! \prod\limits_{r} f\!\left(y^{c}_{ijr}|{s^{c}_{j}} \sum\limits_{t} \tilde{p}_{jt} x^{c}_{itr}\right)\! \right)\!, \end{array} $$

where *r*∈{1*k*,5*k*,10*k*} denotes different window sizes around each genomic loci *i*, $y^{c}_{\textit {ijr}}$ is the read count in heterogeneous input control sample *j*=1,…,*J* in genomic location *i*=1,…,*I* and window size *r* ($x_{\textit {itr}}^{c}$ is defined similarly), and superscript *c* denotes the input control samples. As above, we estimate the cell type specific input control signals in the three windows by maximizing the posterior in Eq. (5) with respect to **x** ^*c*^ using L-BFGS-B.

**Fig. 3:**
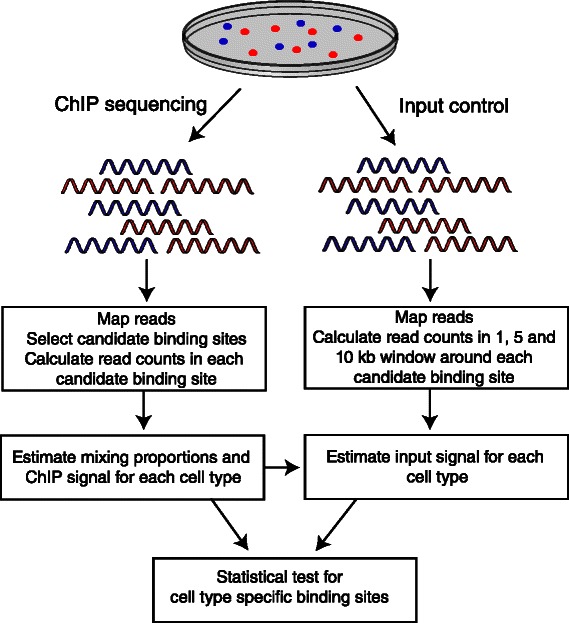
Analysis workflow of the MixChIP method. First, the cell type proportions in each ChIP sample and cell type specific signal are estimated. Second, the estimated proportions are used to deconvolve the signal in the input control samples using three different regions around the candidate binding site regions

Once the cell type specific binding affinities $\tilde {\mathbf {x}}$ and input control signals $\tilde {\mathbf {x}}^{c}$ are estimated, we would like to test for significance of cell type specific binding. We decided to formulate a MACS-like significance test, which uses a dynamic Poisson distribution. In particular, under the null hypothesis of no binding site, the dynamic *λ* parameter in the Poisson distribution for cell type *t* in loci *i* is estimated from the cell type specific signals in the input control, $\tilde {x}^{c}_{it[1k]}$, $\tilde {x}^{c}_{it[5k]}$ and $\tilde {x}^{c}_{it[10k]}$ or from global average of read counts

(6) $$ \tilde{\lambda}_{it}=l_{i} S \max \left(\frac{\tilde{x}^{c}_{it[1k]}}{1000}, \frac{\tilde{x}^{c}_{it[5k]}}{5000}, \frac{\tilde{x}^{c}_{it[10k]}}{10000}, \frac{R^{c}_{global}}{G} \right),  $$

where *l*_*i*_ is the width of the candidate binding site *i* in base pairs in the ChIP sample, *S* is a scaling factor to normalize the sequencing depths between the ChIP and input control sample, $R^{c}_{\textit {global}}$ is the total number of reads in input control experiment and *G* is the size of the genome. As in MACS method, the *p*-value of each $\tilde {x}_{\textit {it}}$ is computed relative to a dynamic Poisson distribution with parameter $\tilde {\lambda }_{\textit {it}}$. The whole analysis workflow of the algorithm is illustrated in Fig. 3.

Computational time depends on the number of sites and samples. To perform optimization 10 times for the simulated data with 8 samples and 23, 160 candidate binding sites takes around 3 h on a standard desktop computer. For breast cancer data set with four samples and 971 candidate binding sites the running time is approximately 2 min.

## Results

### In silico simulated data

#### Cell type specific binding

We estimated cell type specific binding affinity and input control signals in HepG2 and K252 cell lines from eight heterogeneous samples using our model and evaluated statistical significance of the cell type specific binding sites using the dynamic Poisson null model. Hyperparameters were set to values: *w*_0_=4, *a*=0.01, *b*=10000, and **p**_0_=(0.1,0.1,0.2,0.2,0.8,0.8,0.9,0.9) which corresponds to the true cell type proportions or **p**_0_=(0.05,0.2,0.4,0.35,0.8,0.5,0.6,0.7) to show that the method works also when the prior information is not accurate. Accuracy of the cell type specific binding analysis is evaluated using the receiver operating characteristic (ROC) curve, where the binding sites obtained from the pure samples with a stringent *p*-value of <10^−7^ are considered as true binding sites. Because no other computational methods have yet been proposed for cell type specific binding analysis, performance of our probabilistic model is compared to the traditional way of identifying binding sites using multiple samples. First, Poisson test for the read counts (i.e., MACS type of analysis) was performed in each genomic locus in each heterogeneous sample. For each region *i*, the *p*-values were combined by taking the maximum of all individual *p*-values. This corresponds to a test with a null hypothesis that at least one separate null hypothesis is true and the alternative hypothesis that all the alternative hypotheses are true. Computing the maximum of individual *p*-values also corresponds to a commonly used approach where binding sites are defined to be those which are found in all the samples.

**Fig. 4:**
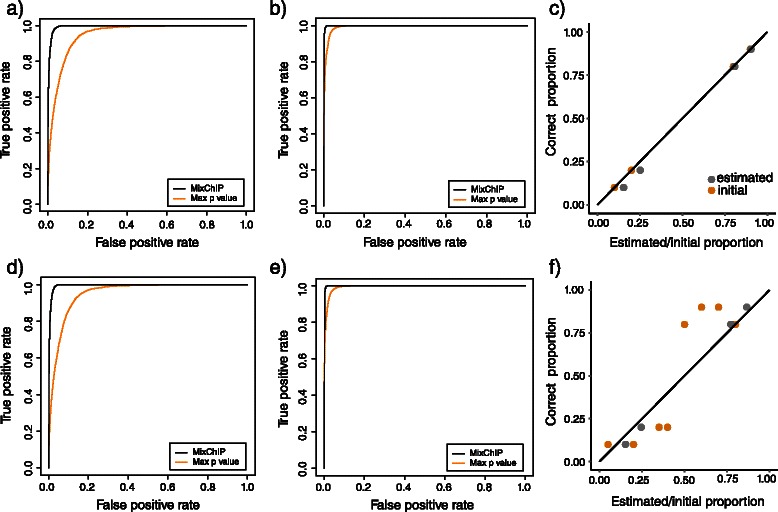
Estimating cell type specific binding affinity and input control signal improves TF binding predictions in the cell types of the interest when compared to the traditional approach where sample heterogeneity is not corrected for. ROC curve of JUND binding predictions for MixChIP (black curve) and combining *p*-values of individual samples by taking the maximum of *p*-values (orange curve) in (**a**) HepG2 and (**b**) K562 cell lines. **c** Estimated proportions (grey dots) of HepG2 cells when true proportions are used as an informative prior (orange dots). **d**–**f** ROCs and the estimated proportions as in (a-c) when priors are intentionally set to differ from the true proportions

The prediction performance in both of the cell lines is shown in Figs. 4(a–b). The detection of JUND binding sites in both of the cells lines is highly specific and sensitive. We also predicted the binding sites in a similar way using all 15 samples (Additional file 1: Figures S1(a–b)). Figs. 4(a–b) and S1(a–b) also show the prediction performance of the traditional maximum *p*-value method. Consistent with the fact that the traditional method is not able to handle sample heterogeneity, we observed that inclusion of samples which have 50 *%* purity decreased its performance (Additional file 1: Figures S1 (c–d)), and accuracy decreased even further if we include all 15 samples (Additional file 1: Figures S1 (e–f)). Consequently, in order to achieve best possible results for the maximum *p*-value method in Figs. 4(a–b) and S1(a–b), we considered only samples which had more than 50 *%* of the cell line of interest when applying the maximum *p*-value method. Overall, our results using simulated heterogeneous ChIP-seq data sets demonstrate that explicitly modeling sample heterogeneity can significantly improve accuracy of binding site detection.

#### Cell type proportions

We tested how sensitive our method is to the choice of prior for cell type proportions **p**. Figs. 4 c) and 4f) show the estimated cell type proportions with different hyperparameter values **p**_0*j*_ but with the same sharpness parameter *w*_0_=4. In Fig. 4 c) the prior means are set exactly to the same locations as the true proportions of the cell types, whereas in Fig. 4 f) prior means are set so that they contain inaccurate information about the proportions. The estimated proportions are close to the true proportions in Fig. 4 c) as expected. However, Fig. 4 f) shows that the probabilistic method is able to infer the cell type proportions from the data, despite the inaccurate information encoded in the prior. Furthermore, using either accurate or inaccurate prior proportions give at the end similar MAP estimates for the cell type proportions as well as for binding affinities, thus resulting in very similar accuracy in binding site detection (compare Figs. 4 (a–b) with (d–e)). We also tested more systematically how different prior information affects the estimated cell type proportions. For a fixed prior sharpness parameter *w*_0_=4, Additional file 1: Figure S2 shows the mean squared error (MSE) between true cell type proportions and initial prior means against MSE between true and estimated proportions. It can be seen that MSE of the estimated values is only a fraction of MSE of initial values. Moreover, different values for the sharpness parameter *w*_0_ did not affect prediction performance either (Additional file 1: Figure S3). Taken together, MixChIP’s performance is not sensitive to small fluctuations in the prior cell type proportions.

#### Effect of the sample size and number of candidate binding sites on binding site identification

**Fig. 5:**
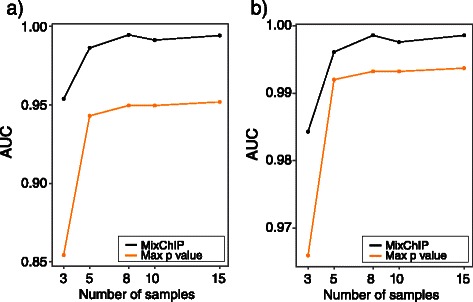
Sample size has only a minor effect for prediction performance. AUC values with different sample sizes using MixChIP (black line) and taking the the maximum of individual *p*-values (orange line) in (**a**) HepG2 and (**b**) K562 cell lines

**Fig. 6:**
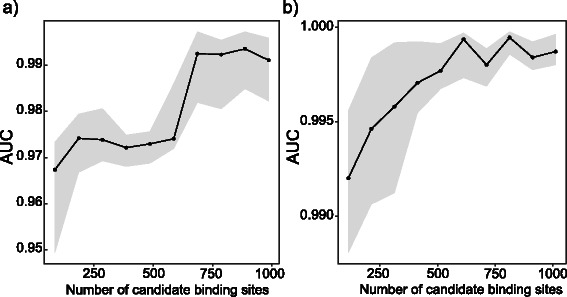
The number of candidate binding sites has a small effect on binding site predictions. AUC values with different number of candidate binding sites for HepG2 (**a**) and K562 (**b**). A subset of candidate binding sites was randomly sampled from the original set of sites and this was repeated 20 times for each subset size. Black line shows the median AUC value among the 20 subsets and grey shaded area shows the 25 and 75 *%* quantiles

Next, we checked how the sample size affects the prediction performance. We identified binding sites using 3, 5, 8, 10 and 15 heterogeneous samples. The mixture proportions of the samples that were used with different sample sizes are shown in Table 2. Again, when applying the maximum *p*-value method, we only used samples which had more than 50 *%* of the cell line where the binding was evaluated. Area under the curve (AUC) values for different sample sizes are shown in Figs. 5(a–b). As sample size increases the prediction performance of the model also increases. However, with only three samples, AUC values in HepG2 and K562 cell lines are as high as 0.95 and 0.98, respectively. The accuracy of the maximum *p*-value method depends more strongly on the sample size and performance is considerably worse on small sample size. Finally, we applied MixChIP to different numbers of candidate binding sites to show that even with only 100 candidate sites, high accuracy can be achieved (see Fig. 6). Collectively, our probabilistic model improves binding site detection for all samples sizes and, importantly, performs well also for small number of samples or candidate binding sites.

**Table 2:** Mixture ratios of HepG2 and K562 cells in samples which were used to test the effect of the sample size

Sample size	Samples
10	10:90 %, 10:90 %, 20:80 %, 20:80 %, 50:50 %, 50:50 %, 80:20 %, 80:20 %, 90:10 %, 90:10 %
8	10:90 %, 10:90 %, 20:80 %, 20:80 %, 80:20 %, 80:20 %, 90:10 %, 90:10 %
5	10:90 %, 20:80 %, 50:50 %, 80:20 %, 90:10 %
3	20:80 %, 50:50 %, 80:20 %

#### Effect of the binding strength

**Fig. 7:**
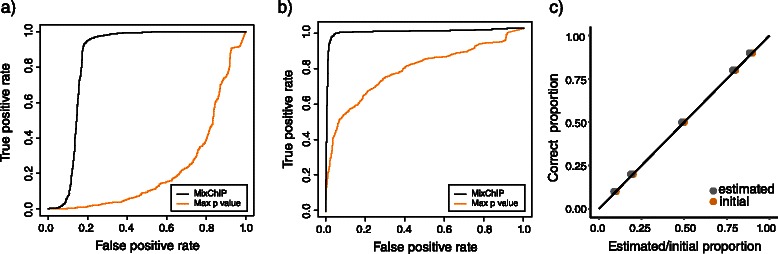
A significant difference in the binding affinity between the cell types can affect the binding prediction accuracy. ROC curve of IRF3 binding predictions in cell lines (**a**) HepG2, (**b**) HeLaS3 and estimated cell type proportions (**c**)

Sometimes the ChIP signal strength between different cell types varies, meaning that on average the binding sites are weaker in one cell type compared to another. This kind of variation can be caused e.g. by altered expression level of the protein of interest and that can affect the cell type specific binding site analysis, especially if the weaker expression and binding sites are in the cell type of interest. To demonstrate this we used ENCODE ChIP-seq data of IRF3 binding in HepG2 and HeLaS3 cell lines. We selected 1248 binding sites in HepG2 cell line and 1300 binding sites in HeLaS3 cell line as true binding sites for the two different cell types and an additional 110 random genomic loci which did not overlap with any of the detected IRF3 binding sites in the two cell lines. The data was preprocessed similarly as the JUND data set. The overall binding strength is stronger in HeLaS3 compared to HepG2 cell line (Additional file 1: Figure S4). Consequently, traditional methods which cannot account for sample heterogeneity primarily detect ChIP-seq signal which originates from the HeLaS3 cell line and, therefore, completely fail to predict IRF3 binding sites in HepG2 (Fig. 7). On the other hand, our probabilistic method is able to account for the sample heterogeneity despite a major difference in the binding signal strength. A small artifact is also seen in the AUC results for the probabilistic model for HepG2 cell line (Fig. 7 a)) as the modeling method incorrectly predicts binding sites in both HepG2 and HelaS3 even though IRF3 is bound to these sites only in HelaS3 cell line.

### Oestrogen receptor- *α* binding data

#### Cell type specific binding

**Fig. 8:**
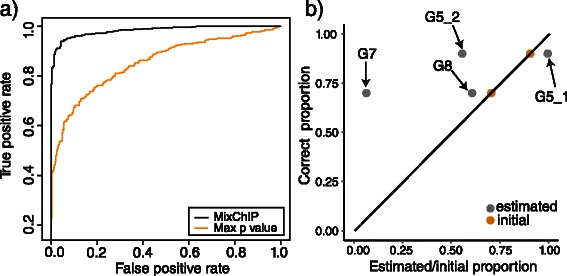
Detecting cell type specific binding sites in primary breast cancer samples using the probabilistic model outperforms the traditional method. **a** ROC curve of ER binding predictions for MixChIP (*b* *l* *a* *c* *k* *c* *u* *r* *v* *e*) and maximum *p*-value method (*o* *r* *a* *n* *g* *e* *c* *u* *r* *v* *e*). **b** The prior (*o* *r* *a* *n* *g* *e* *d* *o* *t* *s*) and estimated proportions (*g* *r* *e* *y* *d* *o* *t* *s*) of breast cancer cells

When detecting ER binding sites using the probabilistic model the hyperparameters were set to the same values as previously with ENCODE data sets. The prediction performance for breast cancer cell specific binding sites is shown as a ROC curve in Fig. 8 a). Again, the performance of MixChIP is compared against the maximum *p*-value method. The probabilistic method outperforms the traditional method. The results in Fig. 8 demonstrate that probabilistic modeling of sample heterogeneity can improve binding site identification also in the case of data from primary cancer biopsies.

#### Cell type proportions

In the breast cancer data set we set the priors to the *a priori* assumed cell type proportions given in [23] with sharpness parameter *w*_0_=4 to have informative but not too strict prior. For two of the samples (G5.1 and G8) the MAP estimates of mixing proportions are close to the prior information, whereas for two of the samples (G5.2 and G7) the estimated mixing proportions are more far away from the priors (Fig. 8 b)). Differences between the prior and posterior estimates may be due to inter individual variation in the ER binding strength which can influence the MAP estimates of the mixture proportions. As an example, sample G7 has on average only 13 reads per binding site, whereas other samples have around 34 reads per binding site. Due to the weaker binding strength, sample G7 has a low estimated breast cancer proportion.

Because the binding strength between the samples varies, we wanted to see how the different scaling factors would affect the results. Instead of using sequencing depth for scaling, we used the binding strength, i.e. the average number of reads in binding sites in each sample. Figure S5 shows that the use of binding strength as a scaling factor helps in correctly estimating the proportions of breast cancer tissue. However, accuracy of cell type specific binding site predictions was decreased. This suggests that there are also other hidden factors, besides sequencing depth, binding strength and the breast cancer cell proportions, that explain the variation between the samples.

## Discussion

In general, transcription factor binding varies between different tissue types. As sequencing costs continue to decrease we will in the future see more ChIP-seq experiments adapted into clinical practice where tissue heterogeneity is a major challenge. In this paper, we have demonstrated a probabilistic method for estimating mixture proportions of different tissue or cell types as well as cell type specific protein binding using heterogeneous ChIP-seq data. Even though the method is applied and benchmarked with samples that are mixtures of two cell types it is straightforward to extend the method to handle more complex mixtures. While computational deconvolution methods based on gene expression data are published regularly, there is a demand to develop *in silico* purification methods for other data types as well.

Using artificially generated mixtures of ENCODE data as well as data from primary breast cancer samples we show that our method outperforms the traditional approach in detecting binding sites from heterogeneous samples. Moreover, accurate predictions can be achieved even with small sample sizes, thus enabling real applications involving heterogeneous samples. Only partial, but not exact, prior information about the true cell type proportions is needed for the deconvolution. Moreover, candidate binding sites can be any sites that show enrichment at least in one of the heterogeneous samples, thus no information about the binding sites in pure cell types is needed. Furthermore, we used rabbit IgG antibody or genomic DNA as input control in our analysis, but in principle any suitable control can be used.

In practice, if the cell type of interest is the major component in heterogeneous samples and a pure ChIP-seq sample from the cell type of interest exists, cell type specific binding sites can be detected from the pure sample using a standard peak finding algorithm. Then, binding sites in each heterogeneous sample can be found in the same way and the sites which were not detected in the pure sample can be discarded as they probably come from the other cell types. However, this kind of approach would neither detect weaker binding sites nor work if the cell type of interest is not the major component in the heterogeneous samples.

In reality, heterogeneous tissue samples can be contain high level of noise. Any standard quality control steps can be used to assess the quality of the ChIP-seq data before applying any deconvolution methods. For instance, manual browser inspection is a good way to evaluate how the experiment and antibody have worked in each sample. As a quantitative measure, one can calculate a fraction of reads that fall into the binding sites [26]. In the ENCODE project samples with more than 1 *%* of reads mapping to the binding sites are labeled as good quality samples [26]. However, when the sample consists of multiple cell types, the quality of the sample can be good even if the fraction of reads in binding sites is less than 1 *%*.

Since binding sites can vary between samples, possible future extensions include e.g. developing a variant of our method which predicts cell type proportions for each sample given binding profiles of a TF in pure cell types, similar to what has been developed for cell type composition estimation in the context of microarray based gene expression data (see e.g. [4, 14]).

## Conclusions

In this work, we propose a probabilistic method, MixChIP, for estimating mixture proportions of different tissue or cell types as well as cell type specific protein binding using heterogeneous ChIP-seq data. We have applied the proposed method to artificially generated mixtures of ENCODE data and data from primary breast cancer samples to show that MixChIP can estimate correct cell type proportions and detects cell type specific TF binding sites more accurately than commonly used approach. We also show that the method is applicable even with small sample sizes and thus can be used in real-life problems.

To our knowledge MixChIP is the first computational deconvolution method designed for ChIP-sequencing data and it can be a valuable tool in analyzing heterogeneous ChIP-seq samples originating, for instance, from tumor biopsy samples.

## Additional file

Additional file 1
**The following additional data are available.** In the Additional file we provide partial derivatives of the log posterior. **(Table S1):** is a table listing ENCODE data samples used in this paper. **(Table S2):** is a table listing breast cancer samples used in the paper. **(Figure S1):** is a figure showing the performance of the method using all the samples and the max *p*-value method when including samples with different amount of purity. **(Figure S2):** is a figure showing MSE between initial cell type proportions and true cell type proportions against MSE between estimated proportions and true proportions. **(Figure S3):** is a figure showing the prediction performance with different values of hyperparameter *w*_0_. **(Figure S4):** is a figure showing the binding strength of IRF3 in HelaS3 and HepG2 cell lines. **(Figure S5):** is a figure showing the prediction performance in the breast cancer data set when different scaling factors are used. (PDF 2541 kb)
