# Supplementary material for: MixChIP: a probabilistic method for cell type specific protein-DNA binding analysis
Source: BMC Bioinformatics. 2015 Dec 24;16:413. doi: 10.1186/s12859-015-0834-3 (PMC4690251; doi:10.1186/s12859-015-0834-3)
Supplement: Additional file 1 — The following additional data are available. In the Additional file we provide partial derivatives of the log posterior. (Table S1): is a table listing ENCODE data samples used in this paper. (Table S2): is a table listing breast cancer samples used in the paper. (Figure S1): is a figure showing the performance of the method using all the samples and the max p-value method when including samples with different amount of purity. (Figure S2): is a figure showing MSE between initial cell type proportions and true cell type proportions against MSE between estimated proportions and true proportions. (Figure S3): is a figure showing the prediction performance with different values of hyperparameter w 0. (Figure S4): is a figure showing the binding strength of IRF3 in HelaS3 and HepG2 cell lines. (Figure S5): is a figure showing the prediction performance in the breast cancer data set when different scaling factors are used. (PDF 2541 kb) [file 12859_2015_834_MOESM1_ESM.pdf]

MixChIP: A probabilistic method for cell type specific protein-DNA binding analysis

Sini Rautio and Harri Lähdesmäki

Department of Computer Science
Aalto University
FI-00076 Aalto, Finland

1 Partial derivatives of posterior

Given data $\mathcal{D}$ and hyperparameters ϕ log posterior can be defined as

$$\log(f(\mathbf{x},\mathbf{p}|\mathcal{D},\phi)) \propto \sum_j \left(\log\frac{1}{B(\alpha)} + \sum_t (\alpha_j - 1) log(p_{jt})\right) + \sum_j \sum_i \left(y_{ij}\log(s_j \sum_t p_{jt}x_{it}) - s_j \sum_t p_{jt}x_{it} - \log(\Gamma(y_{ij}+1))\right), \tag{1}$$

where $B(\alpha) = \frac{\prod_j \Gamma(\alpha_j)}{\Gamma\left(\sum_j \alpha_j\right)}$.

Partial derivatives are

$$\frac{\partial}{\partial x_{it}} = \sum_j \left(\frac{y_{ij}}{s_j \sum_{t'} p_{t'j}x_{t'i}} s_j p_{jt} - s_j p_{jt}\right) \tag{2}$$

$$\frac{\partial}{\partial p_{jt}} = \frac{\alpha_j - 1}{p_{jt}} - \frac{\alpha_T - 1}{1 - \sum_{t'=1}^{T-1} p_{jt'}} + s_j \left(\sum_i \frac{y_{ij}}{s_j \sum_{t'} p_{jt'}x_{it'}}(x_{it} - x_{iT}) - x_{it} + x_{iT}\right), \tag{3}$$

for $t = 1 \ldots T-1$. Note that $p_{jT} = 1 - \sum_{t=1}^{T-1} p_{jt}$.

2 Tables

Table S1: Samples in the ENCODE data set and their matching input controls together with GEO accession names.

Cell type	Antibody	GEO accession name
HepG2	JunD	GSM935649
HepG2	Rabbit-IgG	GSM935604
K256	JunD	GSM935569
K256	Rabbit-IgG	GSM935618

Table S2: Samples in the breast cancer data set, their matching input controls and GEO accession names. Samples G5.1 and G5.2 are from the same patient and they share the same input control.

Patient	Antibody	GEO accession name
G1	ER	GSM798383
G1	none	GSM798406
G2	ER	GSM798384
G2	none	GSM798407
G4	ER	GSM798386
G4	none	GSM798408
G5	ER	GSM798387
G5.1	ER	GSM798388
G5.2	none	GSM798409
G6	ER	GSM798389
G6	none	GSM798410
G7	ER	GSM798390
G7	none	GSM798411
G8	ER	GSM798391
G7	none	GSM798412

3 Figures

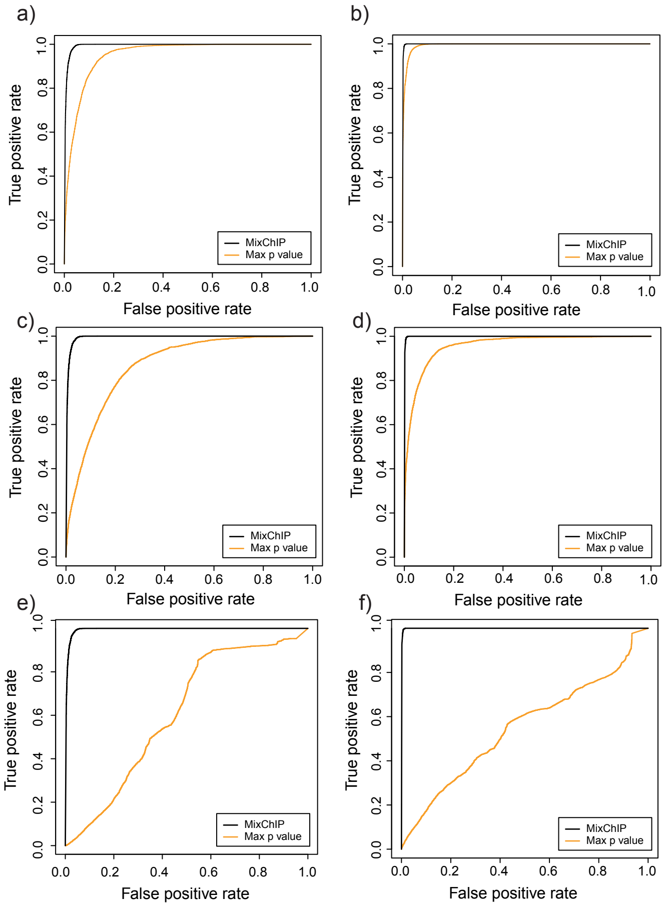

Figure S1: Samples with low purity decrease the performance of the traditional method. ROC curve of JUND binding predictions for using the model with all 15 samples (black curve) and combining p-values of all individual samples by taking the maximum of p-values (orange curve) when including samples which have more than 50% purity (a-b), including only samples which have at least 50% purity (c-d) and using all 15 samples (e-f).

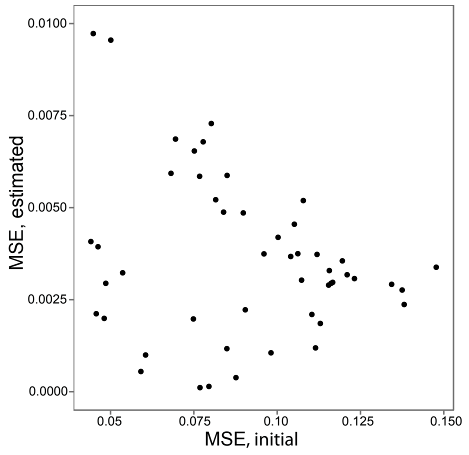

Figure S2: Mean squared error (MSE) between initial cell type proportions and true proportions is multifold compared to MSE of estimated proportions. Y-axis shows MSE between estimated and true proportions and x-axis shows MSE between initial and true proportions.

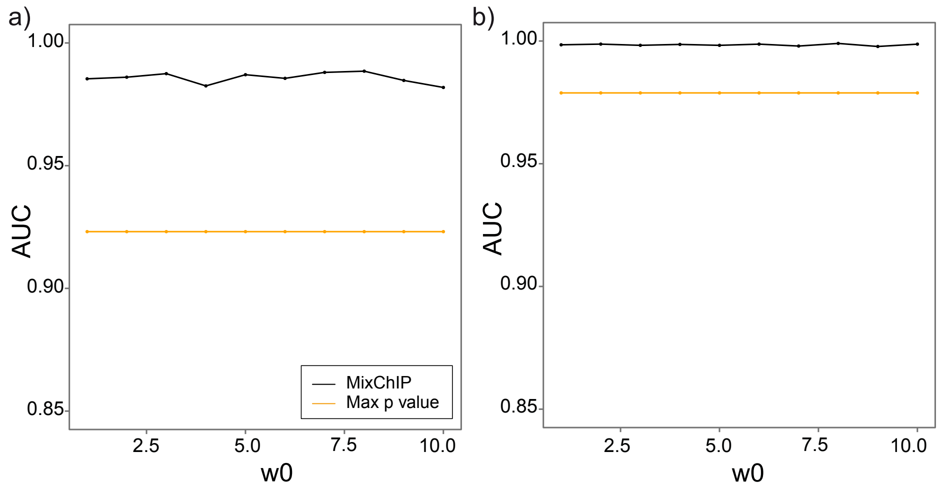

Figure S3: Variance hyperparameter w_0 has a minor effect for binding prediction performance. AUC values with different w_0 values for HepG2 (a) and K256 (b).

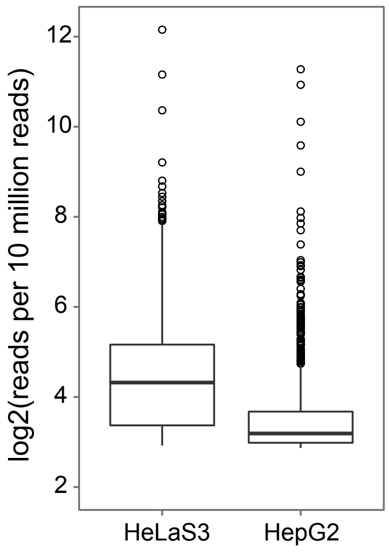

Figure S4: Binding strength of IRF3 is stronger in HeLaS3 cell line compared to HepG2 cell line. Box plots show the read counts per 10 million reads in all IRF3 binding sites.

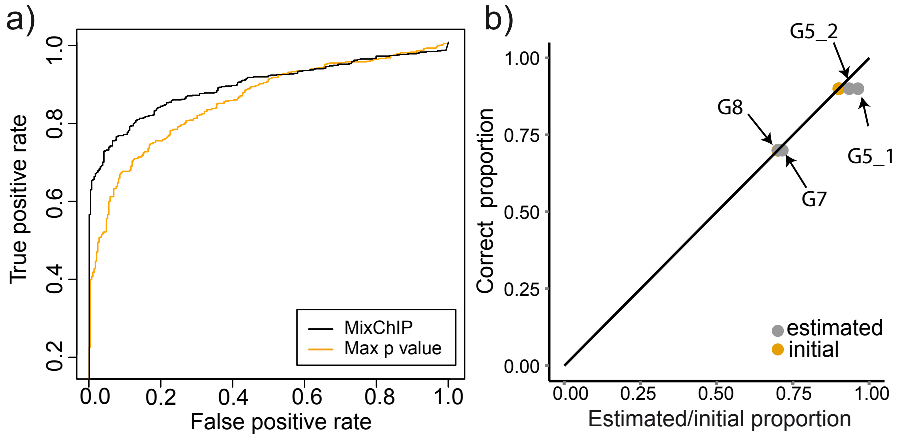

Figure S5: Using a scaling parameter for strength of the binding sites, instead of sequening depths, decreases the prediction performance but estimates the cell type proportions correctly. ROC curve of ER binding site predictions (b) and estimated proportions of breast cancer tissue in each sample (b).
